# Supplementary material for: Identification, characterization, and structure-activity relationship of the ASIC3-selective peptide WRPRFa
Source: Commun Chem. 2025 Dec 12;8:407. doi: 10.1038/s42004-025-01786-7 (PMC12738692; doi:10.1038/s42004-025-01786-7)
Supplement: Supplementary file 2 — Supplementary Information [file 42004_2025_1786_MOESM2_ESM.pdf]

Supplementary Information

Title

Identification, characterization, and structure-activity relationship of the ASIC3-selective peptide WRPRFa

Authors

Chun Chien†, Nien-Du Yang†, Victoria Jiang, Shanti M. Amagasu, and John M. Gilchrist*

Affiliation

Latigo Biotherapeutics, Inc.

Thousand Oaks, United States of America

*Corresponding author: John M. Gilchrist, Latigo Biotherapeutics, Inc., 1300 Rancho Conejo Blvd Suite 305, Thousand Oaks CA 91320, jgilchrist@latigobio.com

†These authors contributed equally

Table S1. Activity of RPRFa derivatives on ASIC3 sustained current. Peptides were tested at 30 μM against ASIC3 on the QPatch II to understand their structure-activity relationship. Arithmetic mean and S.D. are reported for I_{3s}/I_{pk} after peptide treatment. P-value represents multiplicity adjusted p-value from Dunnett's multiple comparisons test, versus RPRFa. Peptides are ordered by descending activity. All were tested on CHO cells.

Peptide	Mean (I_{3s}/I_{pk})	S.D.	N	p-value
RPRFa	0.0476	0.00838	4	
RPRF-COOH	0.00339	0.00259	5	0.0163
RPRF-*p*-nitroanilide	0.387	0.028	5	<0.0001
AARPRFa	-0.0000813	0.0134	4	0.0127
AAAAAARPRFa	-0.00868	0.0311	5	<0.0001
AAAARPRFa	-0.0269	0.0185	4	0.0006
Site 1				
RPR{Cha}a	0.0992	0.0240	4	0.0049
RPRYa	0.0767	0.0458	4	0.366
RPRWa	0.0717	0.0238	5	0.559
RPR{Cpg}a	0.0451	0.0162	7	>0.999
RPRHa	0.00305	0.0174	5	0.0150
RPRDa	0.00163	0.0183	6	0.0067
RPR{Pen}a	-0.000212	0.0126	5	0.0067
RPRAa	-0.00728	0.0130	4	0.0021
Site 2				
RPVFa	0.0188	0.0228	5	0.308
RPTFa	0.00360	0.0263	8	0.006
RPQFa	0.00220	0.00315	5	0.0122
RPKFa	-0.00162	0.00696	4	0.0089
RPIFa	-0.00198	0.00799	5	0.0042
RP{Cit}Fa	-0.00338	0.0169	6	0.0017
RPAFa	-0.00581	0.00848	5	0.0015
RPMFa	-0.0155	0.00711	4	0.0002
Site 3				
RIRFa	0.0569	0.0206	7	>0.999
R{Tic}RFa	0.280	0.0323	6	<0.0001
RWRFa	0.121	0.0216	4	<0.0001
RARFa	0.0271	0.00743	5	0.781
RVRFa	0.00810	0.00413	2	0.238
Site 4				
MPRFa	0.00682	0.0208	6	0.0249
APRFa	0.00220	0.00212	5	0.0122

KPRFa	0.00170	0.0152	5	0.0108
VPRFa	0.000364	0.00536	5	0.0077
{Cit}PRFa	-0.00321	0.00296	4	0.0060
TPRFa	-0.00929	0.0139	4	0.0012
IPRFa	-0.0103	0.0200	4	0.0009

Table S2. Activity of RPRFa derivatives on ASIC3 sustained current. Peptides were tested at 3 µM against ASIC3 on the QPatch II to understand their structure-activity relationship. Arithmetic mean and S.D. are reported for I_{3s}/I_{pk} after peptide treatment. P-value represents multiplicity adjusted p-value from Dunnett's multiple comparisons test, versus RPRFa. Peptides are ordered as they appear in Table S1. All were tested on CHO cells.

Peptide	Mean (I_{3s}/I_{pk})	S.D.	N	p-value
RPRFa	0.00620	0.00104	3	
RPRF-COOH	0.00354	0.00180	4	>0.999
RPRF-*p*-nitroanilide	0.0759	0.0273	5	0.0623
AARPRFa	-0.0230	0.00329	4	0.966
AAAARPRFa	-0.0285	0.0251	4	0.866
AAAAAARPRFa	-0.0172	0.0650	5	0.999
RPR{Cha}a	-0.0123	0.0170	4	>0.999
RPRYa	0.0345	0.0366	4	0.975
RPRWa	0.0163	0.0265	5	>0.999
RPR{Cpg}a	0.00425	0.00925	7	>0.999
RPRHa	-0.00686	0.0141	5	>0.999
RPRDa	-0.0139	0.0157	7	0.999
RPR{Pen}a	-0.00102	0.0122	5	>0.999
RPRAa	-0.00877	0.0148	4	>0.999
RPVFa	0.00634	0.0259	5	>0.999
RPTFa	-0.00490	0.00719	7	>0.999
RPQFa	-0.00112	0.00625	5	>0.999
RPKFa	-0.0211	0.00827	4	0.983
RPIFa	-0.00728	0.00683	6	>0.999
RP{Cit}Fa	-0.0111	0.0190	6	>0.999
RPAFa	-0.0153	0.00791	3	0.999
RPMFa	-0.0333	0.00911	4	0.728
RIRFa	0.0427	0.120	8	0.677
R{Tic}RFa	0.0598	0.0121	6	0.238
RWRFa	0.0165	0.00565	4	>0.999
RARFa	0.00801	0.00472	5	>0.999
RVRFa	-0.00831	0.0177	4	>0.999
MPRFa	-0.00327	0.0212	6	>0.999
APRFa	0.00207	0.00334	5	>0.999

KPRFa	-0.00728	0.0147	5	>0.999
VPRFa	-0.00710	0.00964	4	>0.999
{Cit}PRFa	-0.00171	0.00386	4	>0.999
TPRFa	-0.0141	0.0173	4	1.00
IPRFa	-0.0178	0.0195	4	0.997

Table S3. Activity of WRPRFa derivatives on ASIC3 sustained current. Peptides were tested at 3 μM against ASIC3 on the QPatch II to understand their structure-activity relationship. Geometric mean and geometric S.D. are reported for I_{3s}/I_{pk} after peptide treatment. P-value represents multiplicity adjusted p-value from Dunnett's multiple comparisons test, versus WRPRFa. Peptides are ordered as they appear in Table 1.

Peptide	Geometric mean (I_{3s}/I_{pk})	Geometric S.D. factor	N	p-value
WRPRFa	0.410	1.24	8	
WRPRF-COOH	0.0303	1.07	6	<0.0001
Site 1				
WRPR{Cha}a	0.510	1.08	5	<0.0001
WRPR{Phe(F5)}a	0.168	1.02	5	<0.0001
WRPR(HomoPhe}a	0.240	1.22	4	<0.0001
WRPRWa	0.150	1.18	6	<0.0001
WRPR{Cpg}a	0.277	1.06	4	<0.0001
WRPRIa	0.130	1.10	5	<0.0001
WRPRYa	0.111	1.36	4	<0.0001
WRPR{Tic}a	0.0412	1.16	6	<0.0001
WRPRAa	0.0248	1.25	4	<0.0001
WRPRHa	0.00732	9.83	4	<0.0001
Site 2				
WRP{HomoArg}Fa	0.529	1.09	6	<0.0001
WRP{Arg(Me)}Fa	0.199	1.20	5	<0.0001
WRP{SDMA}Fa	0.100	1.16	6	<0.0001
WRP{ADMA}Fa	0.0612	1.38	4	<0.0001
WRPKFa	0.0129*	0.0100*	4	<0.0001
WRPIFa	0.0168*	0.0539*	7	<0.0001
WRPNFa	0.00879	1.46	5	<0.0001
WRPQFa	0.00859	1.56	4	<0.0001
WRPAFa	-0.00193*	0.00481*	5	<0.0001
WRP{Cit}Fa	0.00444*	0.0110*	5	<0.0001
WRPDFa	0.00402	2.16	5	<0.0001
WRPEFa	0.00309	1.39	4	<0.0001
Site 3				
WRSRFa	0.262	1.26	4	<0.0001
WRARFa	0.239	1.13	6	<0.0001
WR{Tic}RFa	0.167	1.42	4	<0.0001
WRNRFa	0.0823	1.25	4	<0.0001
WRQRFa	0.0736	1.89	7	<0.0001
WRGRFa	0.0447	1.84	7	<0.0001
Site 4				

W{HomoArg}PRFa	0.292	1.12	4	<0.0001
WKPRFa	0.151	1.07	5	<0.0001
WIPRFa	0.0446	1.22	5	<0.0001
WMPRFa	0.0234	1.37	5	<0.0001
W{Cit}PRFa	0.0244	1.25	4	<0.0001
WAPRFa	0.0122	1.96	4	<0.0001
WEPRFa	0.00522	1.24	4	<0.0001
WDPRFa	0.00437	1.17	4	<0.0001
Site 5				
YRPRFa	0.218	1.28	4	<0.0001
FRPRFa	0.243	1.09	4	<0.0001
{HomoPhe}RPRFa	0.138	1.25	4	<0.0001
{Phe(4-NH_2)}RPRFa	0.0988	1.19	4	<0.0001
IRPRFa	0.0770	1.60	4	<0.0001
{Cpg}RPRFa	0.0933	1.14	4	<0.0001
{Cpa}RPRFa	0.0834	1.17	5	<0.0001
{Tic}RPRFa	0.0665	1.15	4	<0.0001
{Cha}RPRFa	0.0667	1.26	4	<0.0001
ARPRFa	0.0254	1.97	5	<0.0001
{Phe(F5)}RPRFa	0.0197	1.71	4	<0.0001
RRPRFa	0.0263	1.36	5	<0.0001
VRPRFa	0.0213	1.33	5	<0.0001
HRPRFa	0.00731	4.15	4	<0.0001
ERPRFa	-0.00418*	0.00269*	4	<0.0001

*arithmetic mean ± S.D.

Supplementary figure legends

Figure S1. RPRFa exhibits a small effect on ASIC1a. Sustained current remaining 10 seconds after peak for ASIC1a (A) and ASIC2a (B) before and after 30 µM RPRFa treatment. ASIC1a was activated by a pH 6.3 stimulus and ASIC2a by a pH 4.0 stimulus. ASIC1a before, I_{10s}/I_{pk}: 0.00803 ×/÷ 1.53 and ASIC1a after, I_{10s}/I_{pk}: 0.0724 ×/÷ 1.36. Significance measured with two-tailed paired ratio t-test. ASIC2a before, I_{10s}/I_{pk}: 0.122 ×/÷ 1.56 and ASIC2a after, I_{10s}/I_{pk}: 0.136 ×/÷ 1.61. Significance measured with two-tailed paired ratio t-test. Data collected from 5 cells for ASIC1a and 4 cells for ASIC2a.

Figure S2. CHO and HEK cells stably expressing ASIC3 have similar gating properties. Comparison of the (A) pH-dependence of activation and (B) desensitization rate for CHO and HEK stable ASIC3 cell lines tested on the QPatch II. In (A), CHO pH_{50}: 6.33, 95% CI: 6.26 to 6.40, and HEK pH_{50}: 6.45, 95% CI: 6.37 to 6.52; 80.1% pH_{50} are different. Data collected from 8 cells for CHO and 8 cells for HEK. In (B), CHO τ: 606 ms ×/÷ 1.16 and HEK τ: 484 ms ×/÷ 1.34; $p = 0.0498$ using unpaired two-tailed t-test. Data collected from 10 cells for CHO and 10 cells for HEK. (C) Standard curve for plasmid titration used in qPCR experiment to quantify expression in HEK ASIC3 stable cell line. Black lines represent semilog fits to standard curves. Red lines show the measured Ct value for ASIC1a and ASIC3 and the interpolated copy number. (D) Copy number for ASIC1a and ASIC3 plotted on log scale. Bars represent arithmetic mean and error bars standard deviation. ASIC3 copy number is 307-fold greater than ASIC1a. (E) Representation of each homomeric or heteromeric channel given ASIC1a:ASIC3::1:307. Percentage assumes equal transcription and random subunit assembly. Homomer frequences calculated by $(a / (a+b))^3$ and heteromers by $(3 * (a / (a+b)))^2 * (b / (a + b)$ where a and b are ASIC1a and ASIC3, or ASIC3 and ASIC1a.

Figure S3. Design of a QPatch II screening assay. (A) diagram of liquid application protocol for the QPatch II peptide screen. Steps represent changes in extracellular pH and red bars application of peptide to the bath. (B) Example screenshot from Sophion

Analyzer software illustrating output of peptide screen on QPatch II platform. (C) Structure of WRPRFa.

Figure S4. WRPRFa shows selectivity against ASIC1a. Sustained current remaining 10 seconds after peak for ASIC1a (A) and ASIC2a (B) before and after 30 μM WRPRFa treatment. ASIC1a was activated by a pH 6.3 stimulus and ASIC2a by a pH 4.0 stimulus. ASIC1a before, I_{3s}/I_{pk}: 0.00456 ×/÷ 6.56 and ASIC1a after, I_{3s}/I_{pk}: 0.0142 ×/÷ 2.41. Significance measured with two-tailed paired ratio t-test. ASIC2a before, I_{3s}/I_{pk}: 0.0903 ×/÷ 2.23 and ASIC2a after, I_{3s}/I_{pk}: 0.0873 ×/÷ 1.93. Significance measured with two-tailed paired ratio t-test. Data collected from 5 cells for ASIC1a and 4 cells for ASIC2a.

Figure S5. Structures of non-canonical amino acids used in the study. Amino acid structure for all non-canonical amino acids

Figure S6. Dearomatization of the site 1 Phe increases peptide potency. Concentration-response curves for four peptides on ASIC3 measured on QPatch II. WRPRFa EC_{50}: 6.31 μM, 95% CI: 5.93 to 6.72 μM; WRPR{Cha}a EC_{50}: 2.33 μM, 95% CI: 2.05 to 2.67 μM; WKPRFa EC_{50}: 9.08 μM, 95% CI: 8.35 to 9.95 μM; FRPRFa EC_{50}: 7.84 μM, 95% CI: 6.71 to 9.39 μM. Data collected from 6 cells for WRPRFa, 6 cells for FRPRFa, 4 cells for WKPRFa, and 5 cells for WRPR{Cha}a.

Figure S7. Further N-terminal extension does not enhance activity. Graph plotting the I_{3s}/I_{pk} for 30 μM FRRFa, PWRPRFa, WWRPRFa, and AWRPRFa. FRRFa I_{3s}/I_{pk}: 0.188 ×/÷ 1.21; PWRPRFa I_{3s}/I_{pk}: 0.610 ×/÷ 1.22; WWRPRFa I_{3s}/I_{pk}: 0.543 ×/÷ 1.05; AWRPRFa I_{3s}/I_{pk}: 0.454 ×/÷ 1.32.

Figure S8. RPRFa induces a small shift in ASIC3 pH sensitivity. (A) Alkaline shift of ASIC3 pH_{50} by 30 μM RPRFa shown in orange. Dotted black line is the WT ASIC3 pH_{50} replotted from Figure 3A. RPRFa pH_{50}: 6.44, 95% CI: 6.39 to 6.50. Data collected from 6 cells. (B) Fold-increase in peak current in response to a pH 6.3 stimulus after treatment with 30 μM WRPRFa. Experiment was performed on the QPatch II.

Figure S9. Time-dependence of recovery from acute desensitization for ASIC3. (A) Channels are activated by pH 5.0 (blue) or 6.3 (black) for 3 seconds and then recovered at pH 8.0 for increasing durations of time. A second test pulse to pH 5.0 or 6.3 measures the fraction of channels recovered. pH 5.0 τ: 2170 ms, 95% CI: 1860 to 2540 ms. pH 6.3 τ: 927 ms, 95% CI: 716 to 1200 ms. Data collected from 8 cells for pH 5.0 and 5 cells for pH 6.3. (B) Protocol (top) and trace (below) for peptide unbinding experiment. Roman numerals indicate different peaks used to calculate sustained current fraction shown in (C). Cells were activated by pH 6.0 for 15 seconds (peak i), followed by a 2 second recovery at pH 8.0, then another 10 seconds at pH 6.0 (peak ii). Cells were recovered at pH 8.0 for 22 seconds, then activated at pH 6.0 again for 15 seconds (peak iii). (C) I_{3s}/I_{pk} for each peak of the experiment shown in (B). Data collected from 5 cells. Significance calculated by Tukey's multiple comparisons test following one-way ANOVA. (D) Fraction of I_{pk} and I_{3s} recovered for peak ii relative to peak i (left) and peak iii relative to peak i (right). Significance was calculated by one sample t-test versus a theoretical mean of 1.

Figure S10. Currents do not recover after successive activation by pH 4.0 (A) Graph showing the fraction of peak current remaining after 10 stimulations (#10) and after a 60 second recovery time (#10 + 60 seconds) does not change. Significance measured with two-tailed paired ratio t-test. (B) Example trace for one of the cells plotted in (A). Data collected from 5 cells.

Figure S11. Summary of the RFamide pharmacophore for ASIC3.

Figure S1

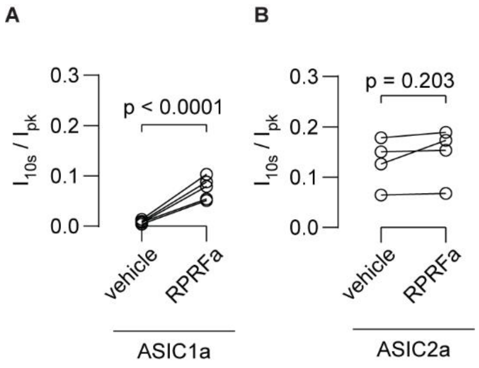
A
B
I_{10s} / I_{pk}
0.3
0.2
0.1
0.0
p < 0.0001
p = 0.203
vehicle
RPRFa
ASIC1a
ASIC2a

Figure S2

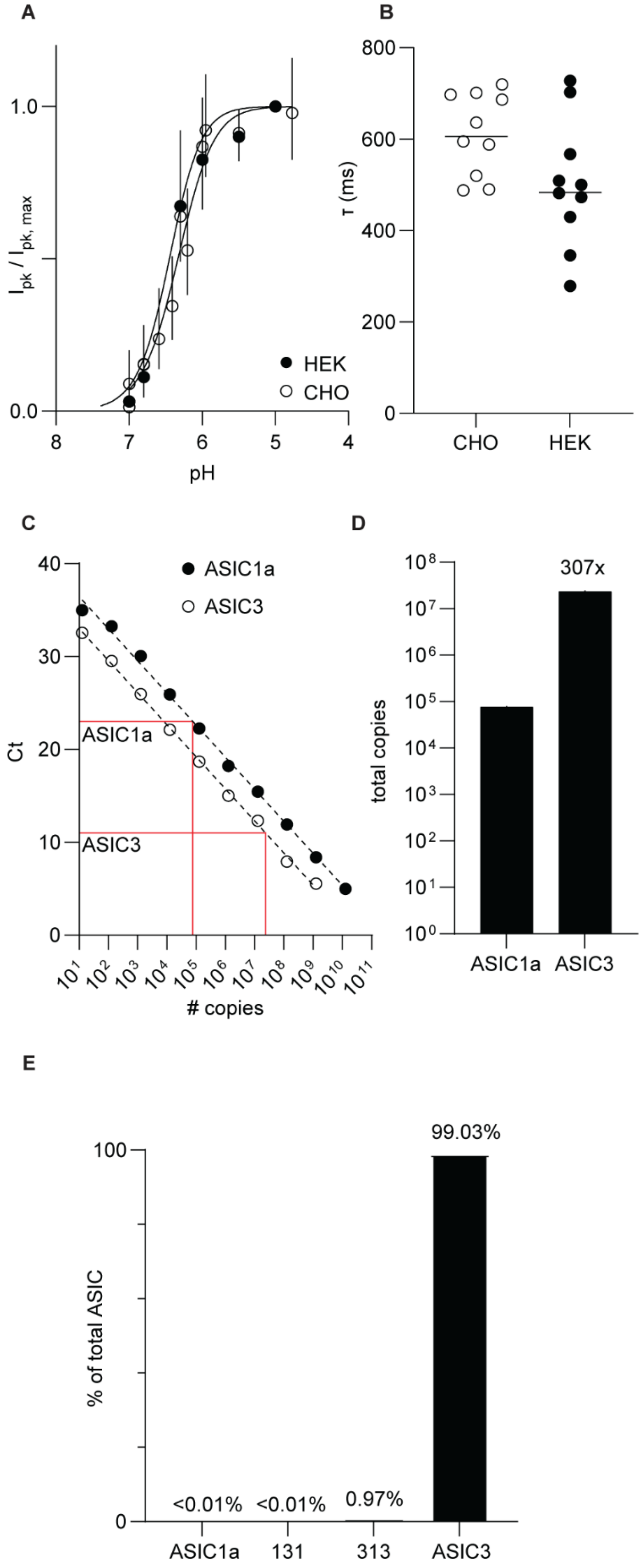

A
B
C
D
E
1.0
0.0
I_{pk} / $I_{pk, max}$
pH
HEK
CHO
τ (ms)
CHO
HEK
Ct
ASIC1a
ASIC3
copies
total copies
307x
% of total ASIC
99.03%
<0.01%
<0.01%
0.97%
ASIC1a
131
313
ASIC3

Figure S3

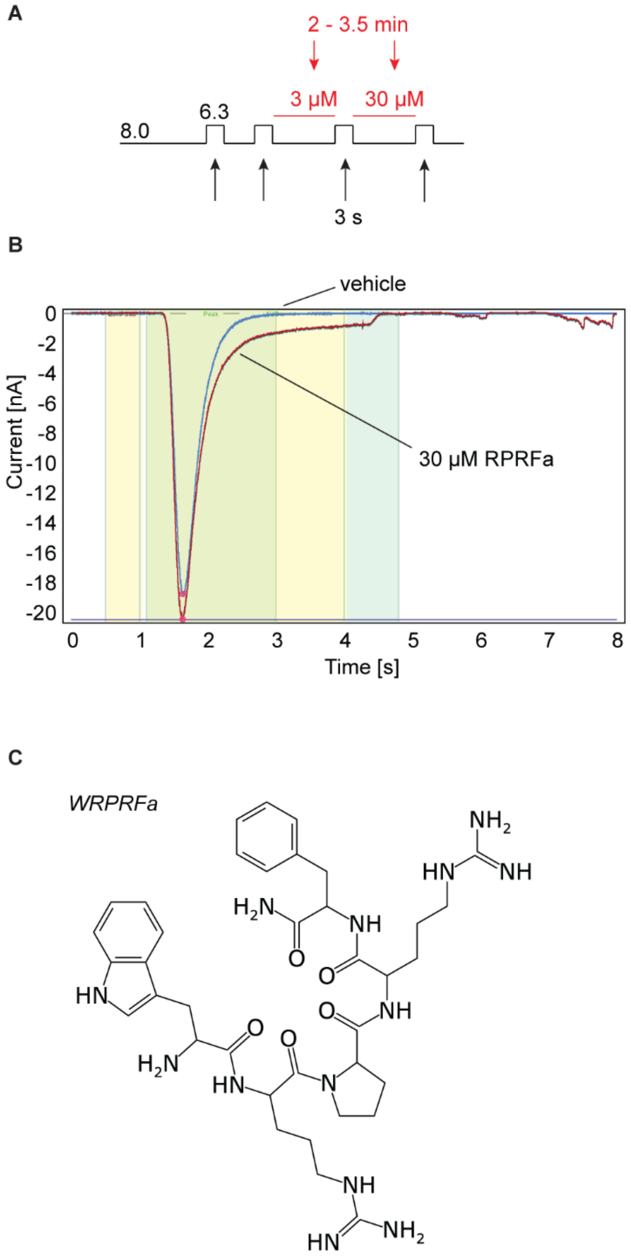
A
2 - 3.5 min
3 μM
30 μM
8.0
6.3
3 s
B
vehicle
30 μM RPRFa
Current [nA]
Time [s]
0
-2
-4
-6
-8
-10
-12
-14
-16
-18
-20
0
1
2
3
4
5
6
7
8
C
WRPRFa

Figure S4

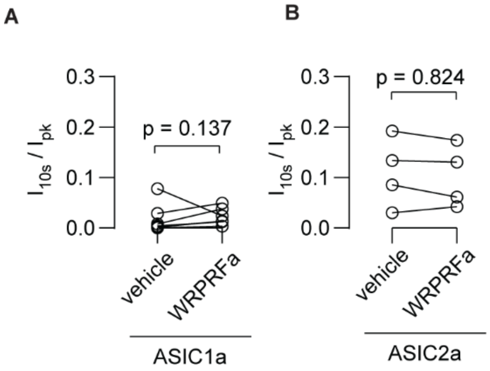
A
B
0.3
0.2
0.1
0.0
I_{10s} / I_{pk}
p = 0.137
vehicle
WRPRFa
ASIC1a
p = 0.824
ASIC2a

Figure S5

HomoArg
homoarginine

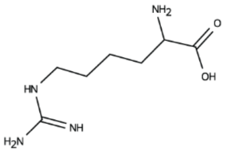

Arg(Me)
methylarginine

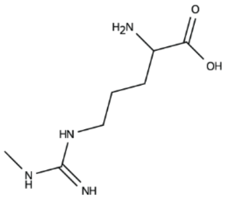

SDMA
dimethylarginine, symmetrical

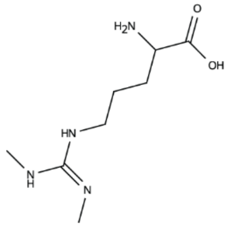

ADMA
dimethylarginine, asymmetrical

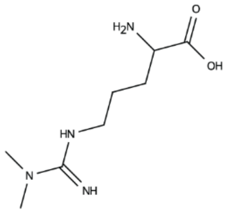

HomoPhe
homophenylalanine

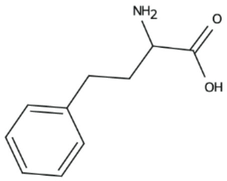

Phe(F5)
pentafluorophenyl alanine

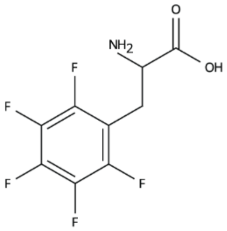

Phe(4-NH_2)
4-amino-phenylalanine

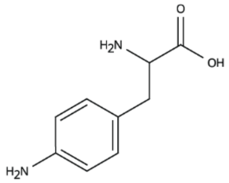

Cha
cyclohexylalanine

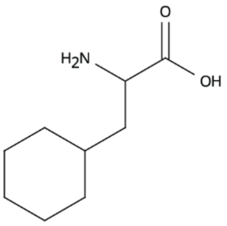

Cpg
cyclopentylglycine

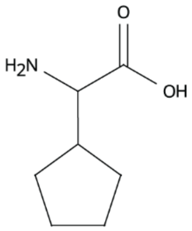

Cpa
cyclopropylalanine

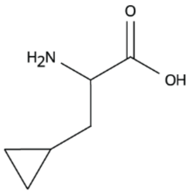

Cit
citrulline

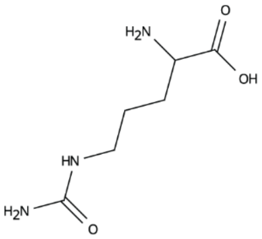

Tic
1, 2, 3, 4-tetrahydroisoquinoline-3-carboxylic acid

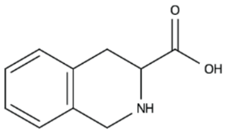

p-na
-para-*nitroanilide*

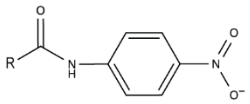

Figure S6

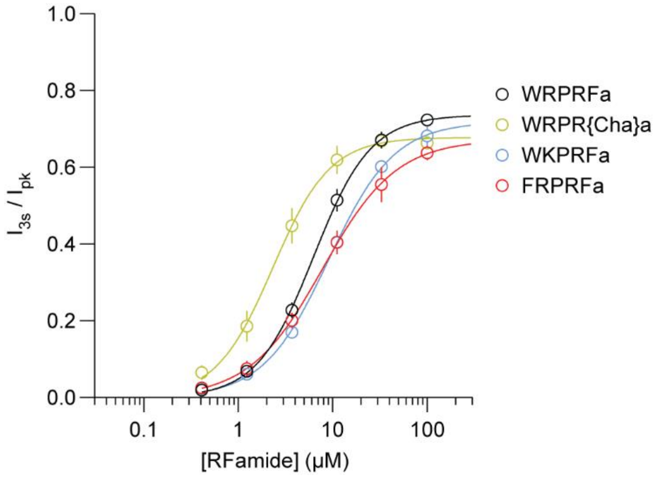

1.0
0.8
0.6
0.4
0.2
0.0
I_{3s} / I_{pk}
0.1
1
10
100
[RFamide] (µM)
WRPRFa
WRPR{Cha}a
WKPRFa
FRPRFa

Figure S7

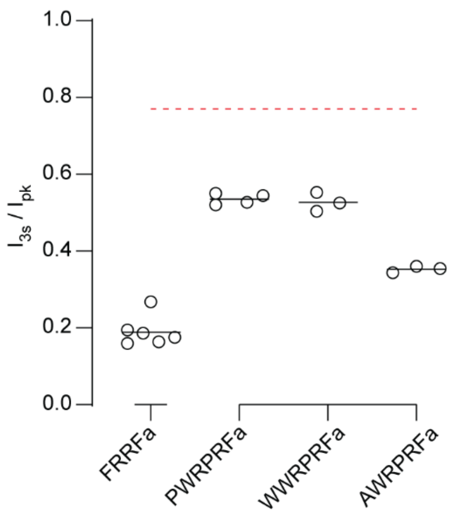

Figure S8

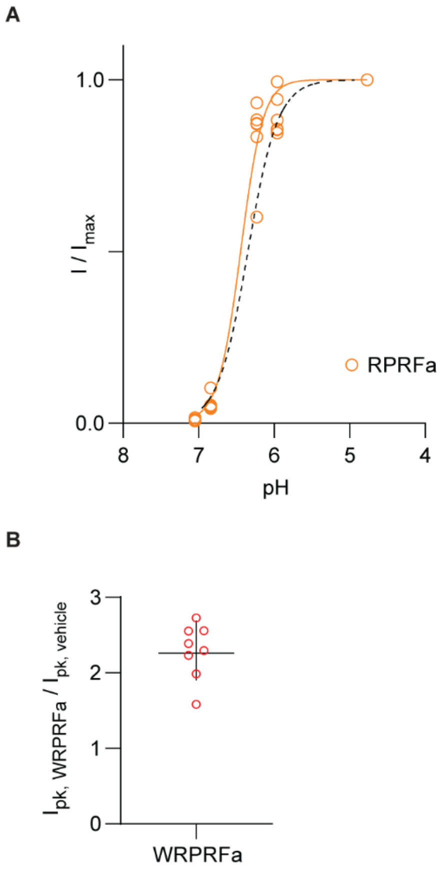
A
1.0
0.0
I / I$_{max}$
RPRFa
8
7
6
5
4
pH
B
3
2
1
0
I$_{pk, WRPRFa}$ / I$_{pk, vehicle}$
WRPRFa

Figure S9

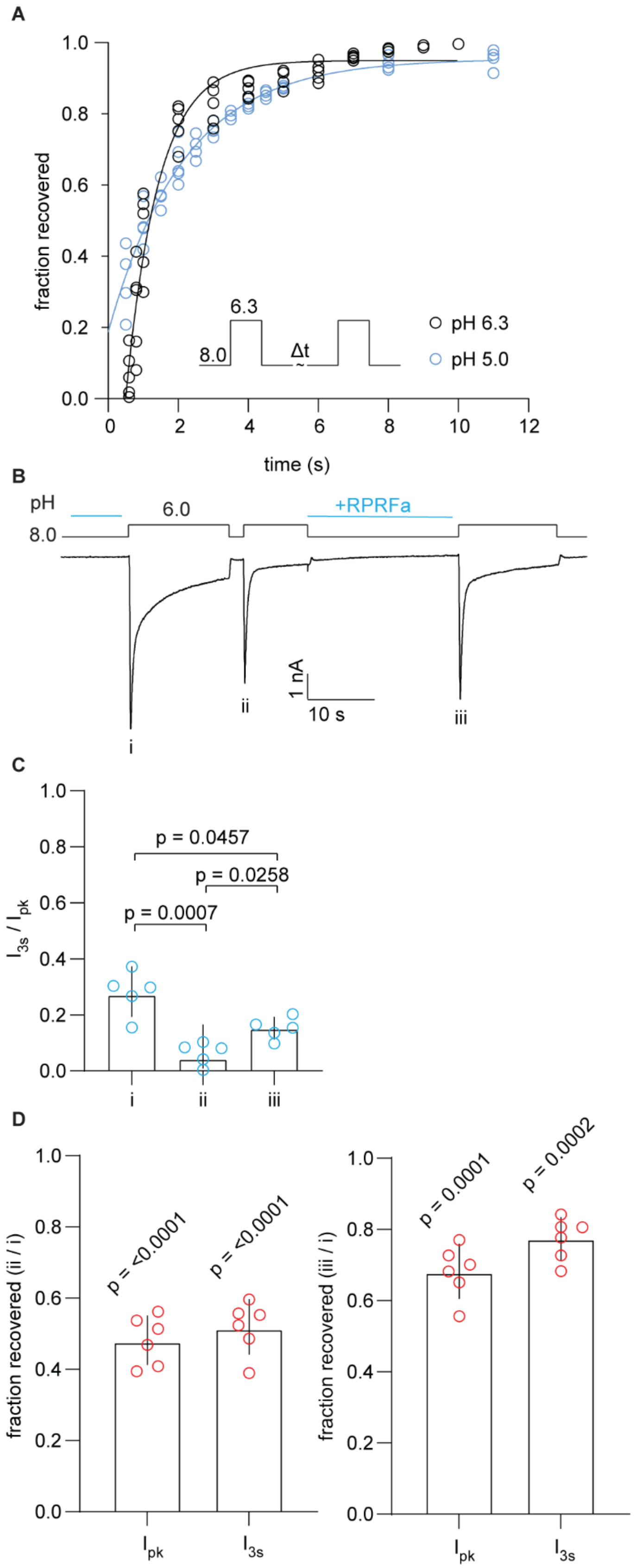
A
1.0
0.8
0.6
0.4
0.2
0.0
fraction recovered
0
2
4
6
8
10
12
time (s)
6.3
8.0
Δt
pH 6.3
pH 5.0
B
pH
6.0
+RPRFa
8.0
1 nA
10 s
i
ii
iii
C
p = 0.0457
p = 0.0258
p = 0.0007
I3s / Ipk
D
fraction recovered (ii / i)
p = <0.0001
p = <0.0001
Ipk
I3s
fraction recovered (iii / i)
p = 0.0001
p = 0.0002

Figure S10

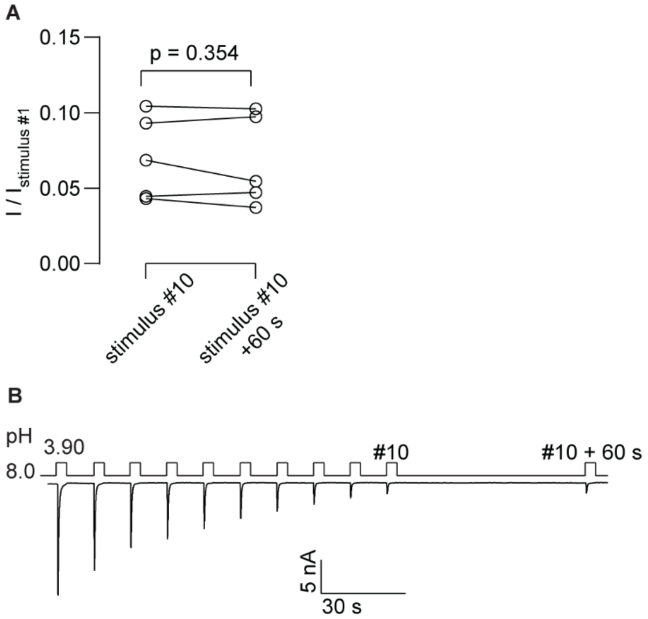
A
0.15
0.10
0.05
0.00
I / I$_{stimulus\ \#1}$
p = 0.354
stimulus #10
stimulus #10
+60 s
B
pH
3.90
8.0
#10
#10 + 60 s
5 nA
30 s

Figure S11

site 5	hydrophobic, flat	W
site 4	cationic, long	R
site 3	small, chiral	P
site 2	cationic, H-bonding	R
site 1	hydrophobic, bulky	F
C-term	amidation	amide
