# Supplementary material for: Identification, characterization, and structure-activity relationship of the ASIC3-selective peptide WRPRFa
Source: Commun Chem. 2025 Dec 12;8:407. doi: 10.1038/s42004-025-01786-7 (PMC12738692; doi:10.1038/s42004-025-01786-7)
Supplement: Supplementary file 3 — Description of Additional Supplementary Files [file 42004_2025_1786_MOESM3_ESM.pdf]

## Description of Additional Supplementary Files:

**File:** Supplementary Data 1

**Description:** Excel file containing data used to generate figures contained in the main text and supplementary information
